# Supplementary figures and images for: Seasonal dynamics of microbial diversity in the rhizosphere of Ulmus pumila L. var. sabulosa in a steppe desert area of Northern China
Source: PeerJ. 2019 Aug 22;7:e7526. doi: 10.7717/peerj.7526 (PMC6708578; doi:10.7717/peerj.7526)

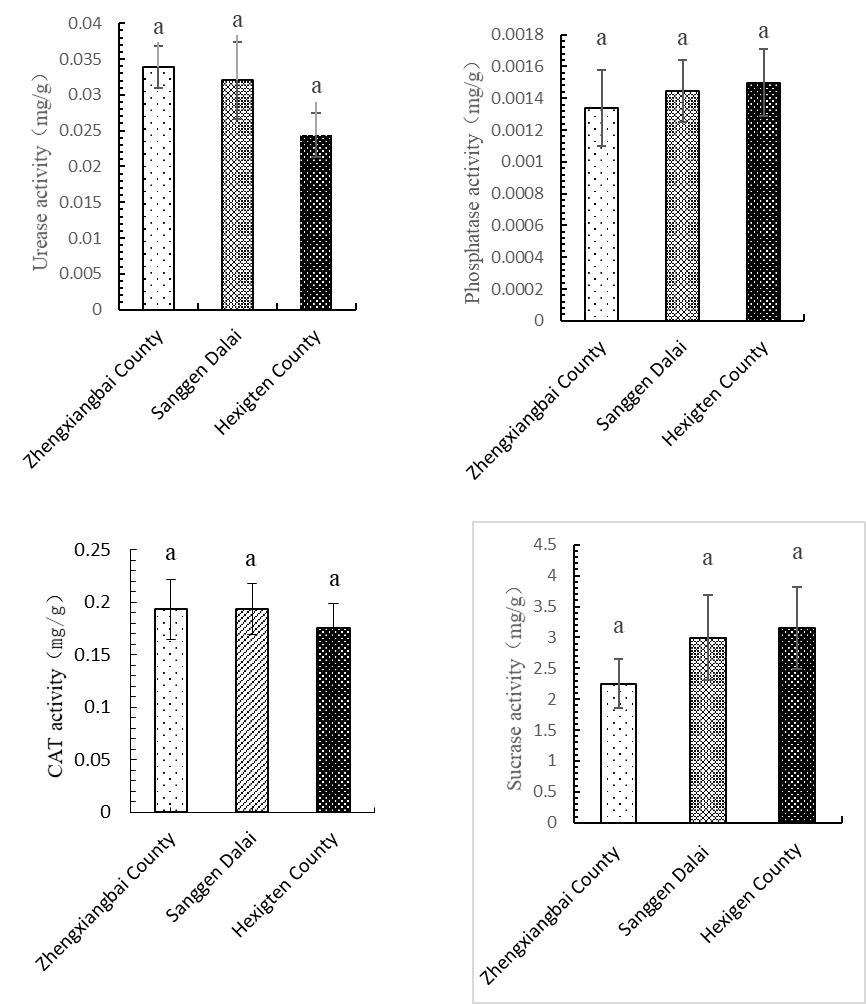

Supplement: Figure S1 — Urease, phosphatase, catalase and sucrase activities of elm rhizosphere soil from different geographic location to prove the spatial heterogeneity of elm rhizosphere soil in the Otindag Sandy Land is low. [file peerj-07-7526-s008.png]

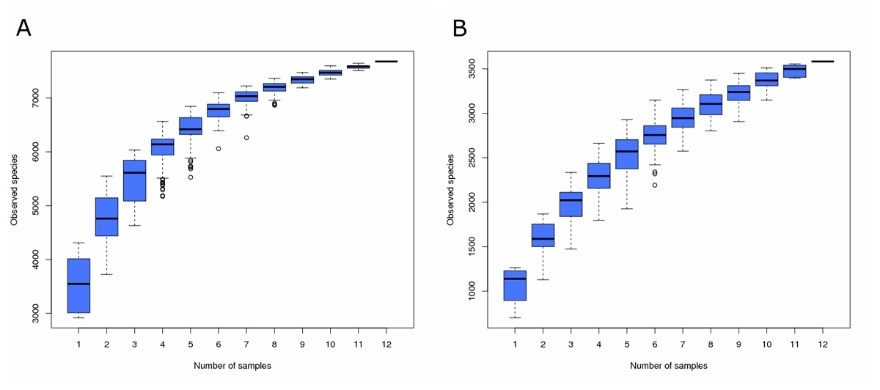

Supplement: Figure S2 — The supplemental material to confirm the sampling number was enough. [file peerj-07-7526-s009.jpg]
