# Supplementary material for: Seasonal dynamics of microbial diversity in the rhizosphere of Ulmus pumila L. var. sabulosa in a steppe desert area of Northern China
Source: PeerJ. 2019 Aug 22;7:e7526. doi: 10.7717/peerj.7526 (PMC6708578; doi:10.7717/peerj.7526)
Supplement: Table S1 — The supplemental material to confirm the spatial heterogeneity of elm rhizosphere soil is low. [file peerj-07-7526-s010.docx]

Table S1. Physical and Chemical Properties of Elm Rhizosphere Soils in Different Geographical Locations

|  | Zhengxiangbai County | Sangen Dalai | Hexigten County |
| --- | --- | --- | --- |
| C/N | 9.07±0.38a | 9.20±0.39a | 10.26±0.47a |
| Total N (mg/100g) | 43.67±6.84a | 57.08±10.23a | 50.5±7.80a |
| Available N (mg/kg) | 57.26±3.99a | 65.13±5.98a | 69.46±4.75a |
| Total P (mg/100g) | 12.90±1.15a | 11.88±0.96a | 10.59±1.26a |
| Available P (mg/kg) | 9.82±1.20a | 5.72±0.53b | 3.55±0.28b |
| Available K (mg/kg) | 182.06±31.45a | 127.20±28.53a | 98.45±14.44a |
| Organic matter (g/kg) | 8.28±1.49a | 10.82±2.03a | 9.45±1.91a |
| pH | 7.80±0.06a | 7.30±0.06b | 7.36±0.07b |
| Moisture content (%) | 3.16±0.16a | 3.41±0.25a | 2.74±0.43a |

Different letters within the same parameter indicate significant differences at the *p* < 0.017 level (Bonferroni corrected results).
