# Supplementary material for: Seasonal dynamics of microbial diversity in the rhizosphere of Ulmus pumila L. var. sabulosa in a steppe desert area of Northern China
Source: PeerJ. 2019 Aug 22;7:e7526. doi: 10.7717/peerj.7526 (PMC6708578; doi:10.7717/peerj.7526)
Supplement: Table S2 — Raw PE: Raw reads number obtained by Illumina HiSeq sequencing platform. Effective Tags: Valid sequences for subsequent analysis after filter chimera. AvgLen: The average length of effective tags. Effective%: The ratio of Effective Tags to Raw PE. It reflected the effectiveness of 16S rRNA gene sequencing. [file peerj-07-7526-s011.docx]

Table S2 Sequencing quality detection of 16S rRNA gene of each sample in different seasons

|  | Raw PE | Effective Tags | AvgLen(nt) | Effective% |
| --- | --- | --- | --- | --- |
| GSP1 | 93,978 | 87,544 | 253 | 93.15 |
| GSP2 | 86,947 | 81,281 | 253 | 93.48 |
| GSP3 | 91,195 | 82,963 | 254 | 90.97 |
| GSP4 | 82,256 | 77,105 | 253 | 93.74 |
| GSU1 | 84,000 | 78,977 | 253 | 94.02 |
| GSU2 | 68,002 | 62,930 | 253 | 92.54 |
| GSU3 | 88,697 | 82,606 | 253 | 93.13 |
| GSU4 | 60,617 | 56,034 | 253 | 92.44 |
| GFA1 | 81,273 | 76,743 | 253 | 94.43 |
| GFA2 | 65,202 | 62,234 | 253 | 95.45 |
| GFA3 | 89,166 | 82.848 | 253 | 92.91 |
| GFA4 | 82,782 | 77,909 | 253 | 94.11 |
| Total | 974,115 | 909,174 | — | — |
| Avg | 81,176 | 75,765 | 253 | 93.00 |
