# Supplementary material for: Seasonal dynamics of microbial diversity in the rhizosphere of Ulmus pumila L. var. sabulosa in a steppe desert area of Northern China
Source: PeerJ. 2019 Aug 22;7:e7526. doi: 10.7717/peerj.7526 (PMC6708578; doi:10.7717/peerj.7526)
Supplement: Table S3 — Raw PE: Raw reads number obtained by Illumina HiSeq sequencing platform. Effective Tags: Valid sequences for subsequent analysis after filter chimera. AvgLen: The average length of effective tags. Effective%: The ratio of Effective Tags to Raw PE. It reflected the effectiveness of ITS region sequencing. [file peerj-07-7526-s012.docx]

Table S3 Sequencing quality detection of ITS sequencs of each sample in different seasons

|  | Raw PE | Effective Tags | AvgLen(nt) | Effective% |
| --- | --- | --- | --- | --- |
| FSP1 | 92,201 | 83,177 | 227 | 90.21 |
| FSP2 | 97,518 | 88,423 | 224 | 90.67 |
| FSP3 | 81,720 | 74,554 | 236 | 91.23 |
| FSP4 | 95,424 | 85,684 | 221 | 89.79 |
| FSU1 | 99,670 | 86,337 | 218 | 86.62 |
| FSU2 | 99,781 | 92,554 | 212 | 92.76 |
| FSU3 | 81,032 | 72,386 | 222 | 89.33 |
| FSU4 | 56,305 | 49,292 | 214 | 87.54 |
| FFA1 | 79,356 | 69,226 | 225 | 87.23 |
| FFA2 | 80,125 | 73,886 | 222 | 92.21 |
| FFA3 | 99,924 | 88,596 | 232 | 88.66 |
| FFA4 | 91,586 | 81,416 | 260 | 88.9 |
| Total | 1,054,642 | 945,531 | — | — |
| Avg | 87,887 | 78,794 | 226 | 90 |
